# A Case of Acute Aortic Dissection Presenting With Neurological Symptoms

**DOI:** 10.7759/cureus.42318

**Published:** 2023-07-23

**Authors:** Mohamed Sheeraz Mohamed Azhar, Mariya Rajesh

**Affiliations:** 1 Internal Medicine, Northampton General Hospital NHS Trust, Northampton, GBR

**Keywords:** aortic dissection (ad), thoracic aortic dissection, atypical presentation of aortic dissection, acute type a aortic dissection, aortic dissection diagnosis, symptoms of aortic dissection

## Abstract

Aortic dissection (AD) is a life-threatening condition that presents with diverse and atypical symptoms, making it challenging to diagnose. We present a case of a woman in her 40s who presented to the emergency department with collapse, right-sided weakness, agitation, and confusion. Despite efforts, she went into cardiac arrest and died before a definitive diagnosis was made. The post-mortem examination revealed hemopericardium due to dissection of the ascending thoracic aorta as the cause of death. This case highlights the difficulty in diagnosing AD and the need for a high index of suspicion, especially in patients presenting with neurological symptoms and risk factors.

## Introduction

Aortic dissection (AD) is a life-threatening condition characterized by the development of a tear in the internal layers of the aorta, leading to blood entering the space between the tunica intima and the media. This can lead to occlusion of the aorta and its branches and ultimately death. AD is considered a rare occurrence, with reported rates of 5 to 30 cases per one million people per year [1]. The condition is often challenging to diagnose due to the wide variation in its presentation, leading to cases being missed or misdiagnosed. Timely diagnosis and intervention are crucial for patient survival. However, due to its diverse clinical presentation and the potential for atypical symptoms, AD can be challenging to recognize, leading to missed diagnoses and adverse outcomes. AD typically presents with a tearing chest pain that radiates to the back with severe hypotension. However, there can also be neurological presentations if the carotid, vertebral, spinal arteries, or vasa nervorum of peripheral nerves are occluded [2]. Neurological presentations include syncope, seizures, spinal cord ischemia, ischaemic neuropathy, and encephalopathy, with the most common presentation being ischemic stroke, often without pain [2]. We present a case of a female in her early 40s who was admitted to the local emergency department with collapse, transient right-side weakness, profound agitation, and confusion on admission. She ultimately went into cardiac arrest and passed away before a definitive diagnosis was made. A post-mortem was performed, and the cause of death was reported as hemopericardium secondary to dissection of the ascending thoracic aorta.

## Case presentation

A female in her early 40s presented to the local emergency department with sudden collapse and marked agitation. She had a medical history of Factor V Leiden deficiency, Stage 2 hypertension, anxiety, and fibromyalgia, as well as a previous appendectomy and hernia repair. Despite these conditions, she had been living independently and was generally in good health.

According to the patient's family, the collapse occurred without any preceding symptoms. They described multiple episodes of loss of consciousness lasting about two minutes each, during which the patient was unable to speak or move. Additionally, she was incontinent of urine and experienced temporary right-sided weakness, which had resolved by the time she arrived at the emergency department. The family was unsure if she had accidentally taken an overdose of any of her medications.

Upon assessment, the patient's vital signs revealed no fever, blood pressure of 90/50 mmHg, oxygen saturation at 99%, and a heart rate of 52 beats per minute (bpm). However, examining the patient was challenging due to her severe agitation and poor cooperation. On admission, her pulse was regular, and a right-sided basal wheeze was noted during the chest examination. Unfortunately, an examination of the heart sounds was not documented. The patient's pupils were equal and reactive to light, and she displayed involuntary movements in all her limbs. Due to her agitation, obtaining a readable ECG trace was not possible. Laboratory investigations, including a full blood count, revealed no significant abnormalities except for mild leukocytosis of 12.0 x 10^9^ g/L. Renal function was near her baseline levels. with an estimated glomerular filtration rate (GFR) of 54 mL/min and creatinine of 107 mmol/L, and the paracetamol/salicylate levels were within the normal range. A blood gas analysis showed metabolic acidosis with a pH of 7.24 and an elevated lactate level of 5.7 mmol/L, which improved and normalized after receiving intravenous fluids for hydration.

**Table 1:** Laboratory Findings Mild leukocytosis but otherwise no significantly deranged values. GFR: glomerular filtration rate.

Investigation	Result	Reference Range
Hemoglobin	133	120–150 g/L
White cell count	12.0	4.0–10.0 10^9^/L
Estimated GFR	54	mL/min
Sodium	144	133–146 mmol/L
Potassium	3.9	3.5–5.5 mmol/L
Urea	4.4	2.5–7.8 mmol/L
Creatinine	107	45–84 umol/L
Glucose	5.9	mmol/L
Creatinine kinase	90	25–200 IU/L
C-reactive protein	1	0–5 mg/L

It is worth noting that the patient had previously presented to the emergency department three months ago with a severe headache and transient black spots in her visual fields. During this admission, she displayed severe agitation and hemiballism despite treatment with benzodiazepines. She underwent investigations for acute ischemic stroke and required sedation with propofol for imaging due to her agitation. However, a non-contrast head CT scan did not reveal any acute intracranial pathology.

**Figure 1:**
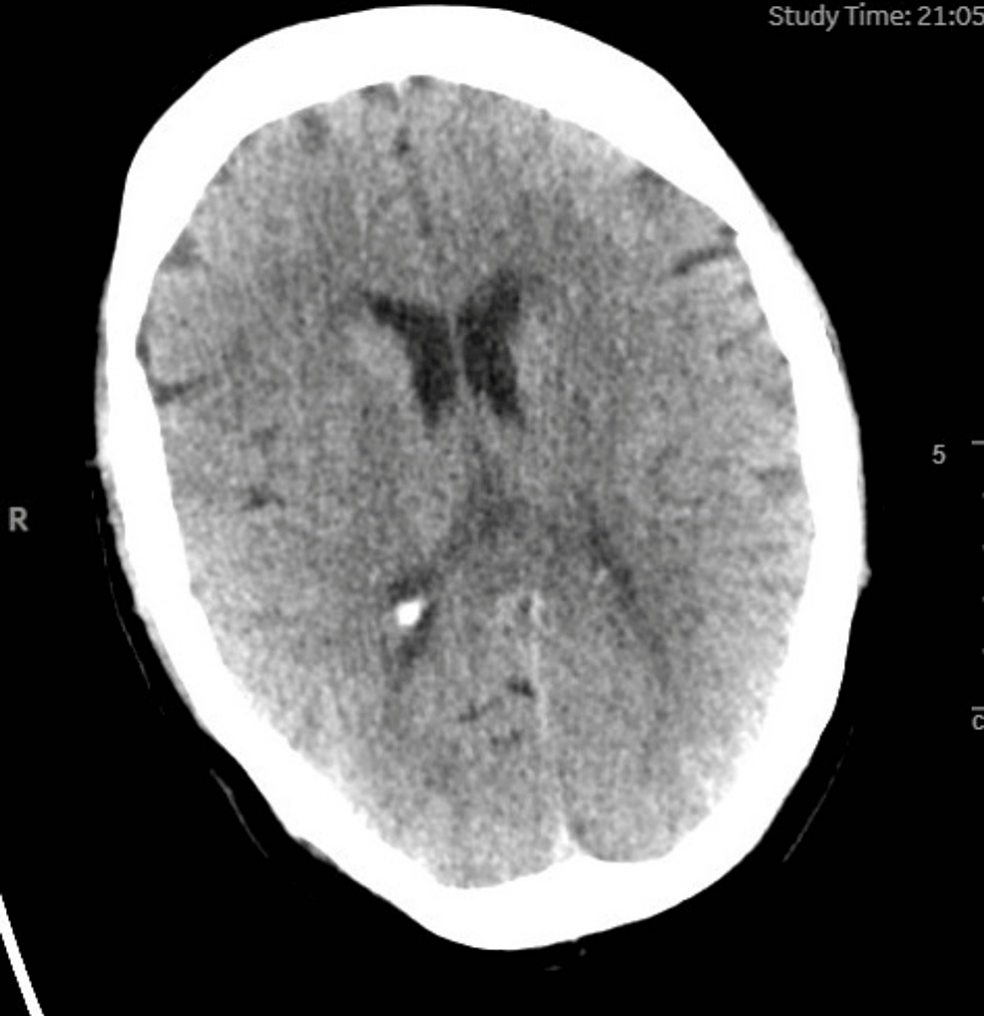
Non-contrast CT head demonstrating no abnormalities

Subsequently, she was empirically treated for suspected meningoencephalitis with intravenous ceftriaxone and acyclovir due to a slight elevation in white blood cells, ongoing confusion, and neurological symptoms. Additional sedation with propofol was required to perform a lumbar puncture, during which the patient became unresponsive and hypoxic, and she experienced pulseless electrical activity (PEA) cardiac arrest. The bedside echocardiogram and ultrasound showed no evidence of tamponade, pulmonary embolism, or pneumothorax. Despite 16 cycles of cardiopulmonary resuscitation (CPR) and multiple boluses of adrenaline, no reversible factors were identified, and the decision was made to cease CPR. The patient passed away peacefully.

The subsequent autopsy revealed the underlying cause of death to be hemopericardium resulting from a 30-mm-length dissection of the ascending thoracic aorta. The autopsy reported that the pericardium contained 250 mL of blood and that the heart was structurally normal but did not mention any extension of the dissection into the major branches of the aorta. The plain CT head of the patient was also reviewed with the reporting radiologist who couldn't identify any evidence of the dissection extending into the arteries in the neck; however, he commented that the images from a plain CT head are not optimal for this purpose.

## Discussion

The presented case report describes the clinical presentation and unfortunate outcome of a female patient in her early 40s who presented to the emergency department with collapse and marked agitation. The case highlights several important aspects, including the challenge of diagnosing AD, the significance of considering aortic pathology in patients with neurological symptoms, and the need for thorough investigations to determine the underlying cause. Diagnosing AD can be challenging due to its diverse and atypical clinical presentation. In this case, the patient's collapse and agitation initially led to a broad differential diagnosis, including neurological causes. However, the missed diagnosis of ascending thoracic AD demonstrates the need for maintaining a high index of suspicion for this life-threatening condition. The presence of risk factors such as hypertension should have raised concerns and prompted further evaluation. Although there is a lot of literature on AD, there are also reports of a significant number of missed cases in the emergency department [3]. AD is broadly categorized into two classifications, and the most frequently used classification system is the Stanford classification of AD. The Debakey classification system is the other one. The Stanford system classifies AD into Type A and Type B. Type A involves the ascending aorta and/or aortic arch. Type B involves the descending aorta, originating distal to the left subclavian artery. The case presented here is a case of Type A AD with the involvement of ascending thoracic aorta. Mortality risk for acute Type A AD has a rate of 0.5% per hour and 23.7% in 48 hours [4]. Prompt recognition and accurate diagnosis are crucial to initiating appropriate management, such as surgical intervention. Missed or delayed diagnosis of AD can have catastrophic consequences, as exemplified in this case.

Furthermore, the neurological symptoms experienced by the patient, including loss of consciousness, right-sided weakness, and involuntary limb movements, highlight the potential involvement of the carotid or vertebral arteries due to compromised blood flow. The neurological manifestations can be atypical and may not include the classic tearing chest pain associated with AD. This case emphasizes the importance of considering aortic pathology even in the absence of characteristic symptoms, especially when there are risk factors or neurological presentations. A few case reports have been published in the literature that describes patients presenting with neurological deficit including transient hemiplegia [5] and seizures [6]. Gaul et al. [2] report that 30% of their patients presented with neurological deficits as the first sign of AD. 

A similar case to our patient was published in 2017 that describes a Type A AD dissection in a 55-year-old male presenting initially with syncope and intermittent convulsions [7]. However, the patient in the above case report was able to give a prodromal history prior to collapse whereas our patient remained confused and agitated throughout her stay in the Emergency Department, thereby making us unable to obtain to get a comprehensive history of presenting complaint. The need for sedation during imaging and lumbar puncture due to the patient's severe agitation further complicated the evaluation. Another similar case that has been published in the literature in 2021 describes syncope as the initial presentation of a 49-year-old male with a background history of hypertension who was subsequently diagnosed with Stanford Type A AD with a poor clinical outcome [8].

The diagnostic process in this case included prior presentations of severe headache, visual disturbances, and neurological symptoms, leading to investigations for acute stroke and suspected meningoencephalitis. Despite the extensive workup, the diagnosis of ascending thoracic AD was missed until the post-mortem examination revealed the cause of death as hemopericardium secondary to AD. 

This case underscores the importance of a systematic and thorough approach to diagnosis, especially in complex cases with multiple presenting symptoms and risk factors. It serves as a reminder that even in the absence of characteristic symptoms, clinicians should consider AD as a potential diagnosis in patients with collapse, neurological symptoms, and risk factors. Early recognition and prompt intervention are crucial to improving patient outcomes in AD cases.

## Conclusions

This case emphasizes the importance of maintaining a high index of suspicion for ascending thoracic AD, particularly in patients presenting with acute collapse and neurological abnormalities including altered mental status, syncope, seizures, and ischemic stroke, which can all occur if blood flow to specific arteries is compromised. Being aware of these diverse presentations is essential for prompt recognition and accurate diagnosis of AD, thereby avoiding delays in treatment and potential adverse outcomes. Therefore, healthcare providers should prioritize timely imaging studies, such as CT angiography or echocardiography, to confirm the diagnosis and should facilitate prompt initiation of appropriate management, such as surgical intervention if necessary. Missed opportunities in the physical examination and timely performance of basic imaging, like a chest X-ray, on this patient, could have potentially prevented the missed or delayed diagnosis. We intend to share the lessons learned in this case with the reader.
